# Supplementary material for: Protective Efficacy of the Epitope-Conjugated Antigen N-Tc52/TSkb20 in Mitigating Trypanosoma cruzi Infection through CD8+ T-Cells and IFNγ Responses
Source: Vaccines (Basel). 2024 Jun 4;12(6):621. doi: 10.3390/vaccines12060621 (PMC11209121; doi:10.3390/vaccines12060621)
Supplement: Supplementary file 1 [file vaccines-12-00621-s001.zip › vaccines-2981536-supplementary.pdf]

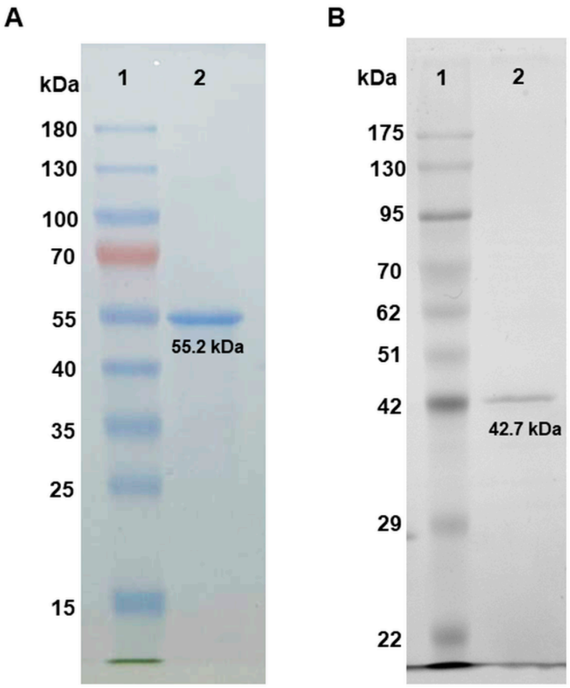

Figure S1. Electrophoretic analysis of the pure rTc52 and rTS proteins in 12% SDS-acrylamide gels. **(A)** lane 1, molecular weight marker PageRuler Prestained Protein Ladder (Thermo Scientific); lane 2, Tc52 protein with a theoretical weight of 55.2 kDa. **(B)** lane 1, PiNK Plus Prestained Protein Ladder (Genbiotech); lane 2, TS protein with a theoretical weight of 42.7 kDa.
